# G-quadruplex DNA contributes to RNA polymerase II-mediated 3D chromatin architecture

**DOI:** 10.1093/nar/gkad588

**Published:** 2023-07-10

**Authors:** Jun Yuan, Xiaomei He, Yinsheng Wang

**Affiliations:** Environmental Toxicology Graduate Program, University of California, Riverside, Riverside, CA 92521-0403, USA; Department of Chemistry, University of California, Riverside, Riverside, CA 92521-0403, USA; Environmental Toxicology Graduate Program, University of California, Riverside, Riverside, CA 92521-0403, USA; Department of Chemistry, University of California, Riverside, Riverside, CA 92521-0403, USA

## Abstract

High-order chromatin organization plays an important role in biological processes and disease development. Previous studies revealed a widespread occurrence of guanine quadruplex (G4) structures in the human genome, with enrichment in gene regulatory regions, especially in promoters. However, it remains unclear whether G4 structures contribute to RNA polymerase II (RNAPII)-mediated long-range DNA interactions and transcription activity. In this study, we conducted an intuitive overlapping analysis of previously published RNAPII ChIA-PET (chromatin interaction analysis with paired-end tag) and BG4 ChIP-seq (chromatin immunoprecipitation followed by sequencing using a G4 structure-specific antibody) data. We observed a strong positive correlation between RNAPII-linked DNA loops and G4 structures in chromatin. Additionally, our RNAPII HiChIP-seq (*in situ* Hi-C followed by ChIP-seq) results showed that treatment of HepG2 cells with pyridostatin (PDS), a small-molecule G4-binding ligand, could diminish RNAPII-linked long-range DNA contacts, with more pronounced diminutions being observed for those contacts involving G4 structure loci. RNA sequencing data revealed that PDS treatment modulates the expression of not only genes with G4 structures in their promoters, but also those with promoters being connected with distal G4s through RNAPII-linked long-range DNA interactions. Together, our data substantiate the function of DNA G4s in RNAPII-associated DNA looping and transcription regulation.

## Graphical Abstract

**Graphical Abstract:**
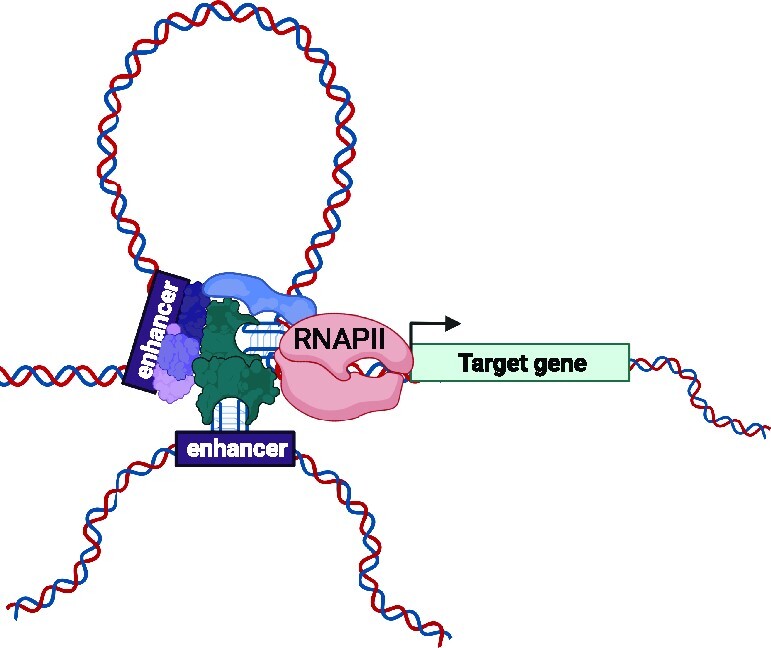

## INTRODUCTION

The DNA guanine quadruplexes (G4s) are four-stranded secondary structures that form in guanine-rich regions of DNA (1,2). G4 structures arise from stacking of at least three layers of G tetrads, where four Gs are held together through hydrogen bonding and a monovalent cation, in the order of K^+^ > Na^+^ > Li^+^ (3). The development of a G4 structure-specific antibody (BG4) enabled probing for the presence of G4 structures in chromatin (4), where BG4 ChIP-seq (chromatin immunoprecipitation followed by sequencing using BG4) results unveiled the enrichment of G4s in regulatory regions of the genome and the presence of distinct G4 landscapes in different cell lines (5,6).

A number of studies revealed the important roles of G4s in various cellular processes, including telomere maintenance (7,8), DNA replication (9), DNA damage (10) and transcription regulation (11–13). Integrative analysis of ChIP-seq data of transcription factors and G4 structures underscored G4s as binding hubs for transcription factors in cells (14). In addition, multiple proteins were shown to bind directly to G4 structures *in vitro* (13,15–19).

Another important element of gene regulation is 3D chromatin architecture, where the nucleus of mammalian cells is highly compacted and organized in a hierarchical fashion ranging from A–B compartment to enhancer–promoter (E–P) contacts (20). Extensive studies have been conducted to investigate the functions of E–P interactions in transcription regulation and their potential contributions to disease development (21–24). In this vein, multiple methods have been developed to profile the detailed 3D organizations of the human genome, including 3C (chromosome conformation capture), 4C (circular chromosome conformation capture), 5C (chromosome conformation capture carbon copy), Hi-C and HiChIP-seq (*in situ* Hi-C followed by ChIP-seq)/ChIA-PET (chromatin interaction analysis with paired-end tag)/PLAC-seq (proximity ligation-assisted ChIP-seq) (25). Among these methods, HiChIP-seq/ChIA-PET/PLAC-seq can detect specific protein-mediated long-range DNA interactions and provide important information about how individual proteins modulate high-order genome organization. Bioinformatic analysis showed the enrichment of G4 structures in topologically associating domain (TAD) boundaries and in E–P interactions, indicating the role of G4s in high-order chromatin organization (26). Moreover, a recent study revealed that Yin-Yang 1 (YY1), which is known to dimerize and enable E–P interactions (27), is able to interact with G4 DNA at high affinity (13). Further HiChIP-seq assay substantiated a YY1-mediated, G4-dependent DNA looping (13). Although multiple studies suggest a role of G4s in distal gene regulation, not much is known about the detailed mechanisms through which G4 structures modulate long-range DNA interactions in cells.

Here, by conducting an overlapping-based analysis of ChIA-PET data in publicly available Encyclopedia of DNA Elements (ENCODE) database (28), we identified a strong correlation between G4 structures in chromatin and RNA polymerase II (RNAPII)-mediated long-range DNA interactions. We also observed that treatment of cells with pyridostatin (PDS), a small-molecule G4-binding ligand, led to more marked decreases in RNAPII-mediated DNA looping at sites with G4 structures than those without. In addition, genome-wide association analysis between ChIA-PET/HiChIP-seq and RNA sequencing (RNA-seq) data provided a comprehensive understanding about transcription regulation mediated by the interplay of G4 structure and RNAPII-mediated DNA looping. Moreover, we showed that enhancer G4s modulate the expression of *AKR1C* (aldo-keto reductase family 1C) family genes in HepG2 cells.

## MATERIALS AND METHODS

### Cell lines

HepG2 human hepatocellular carcinoma cells were cultured in Dulbecco’s modified Eagle’s medium (Life Technologies) containing 10% fetal bovine serum (Invitrogen) and 1% penicillin and streptomycin (Invitrogen). K562 human chronic myelogenous leukemia cells were cultured in RPMI 1640 medium (Life Technologies) containing 10% fetal bovine serum (Invitrogen) and 1% penicillin and streptomycin (Invitrogen). The cells were maintained at 37°C in an incubator containing 5% CO_2_ and the cells were tested to be free of mycoplasma contamination using e-Myco PCR Detection Kits (Bulldog Bio).

### Cell viability assay

Cell viability was examined using Cell Counting Kit-8 (CCK-8, Dojindo) according to the manufacturer’s recommended procedures, where a 100 μl suspension containing 5000 HepG2 cells was plated in each well of a 96-well plate 1 day prior to treatment. Ten microliters of the indicated concentrations of PDS were added to each well, and the cells were incubated for 24 h. After the incubation, 10 μl of CCK-8 solution was added to each well, and absorbance at 460 nm was recorded 3 h later with a BioTek Synergy H1 microplate reader (Agilent Technologies).

### Bioinformatic analysis

ChIA-PET datasets were retrieved through the ENCODE portal under assay title ‘ChIA-PET’, target of assay ‘POLR2A’ and biosample ‘K562’ or ‘HepG2’. POLR2A ChIP-seq datasets were also downloaded from the ENCODE portal under assay title ‘ChIP-seq’. Bedpe files in GRCh38 assembly were downloaded for overlapping analysis and contact matrix hic files were used for visualization. BG4 ChIP-seq raw data for HepG2 and K562 cells were obtained from Sequence Read Archive with the accession number of PRNJ60617 (14). BG4 ChIP-seq data were processed following previously published procedures in GRCh38 assembly (14). Overlapping percentages between RNAPII-linked long-range DNA interactions and G4s were calculated using bedtools pairToBed command with different -type parameters (29). One to multiple overlaps were combined accordingly. Bedpe files were split into two anchors and deduplicated to produce loop anchors. POLR2A ChIP-seq narrowPeak files were overlapped with loop anchors to obtain POLR2A binding sites with or without long-range interactions. Overlap between peaks was calculated using bedtools intersect command. Monte Carlo simulation was conducted by randomly shuffling peak file in target regions using bedtools shuffle command.

### HiChIP and data analysis

HiChIP was performed as previously described (30). Ten million HepG2 cells were mock-treated (with sterilized water) or treated with 20 μM PDS for 24 h before cross-linking with a freshly prepared 1% formaldehyde solution at room temperature for 10 min. After quenching with glycine at a final concentration of 125 mM for 10 min, the cells were washed several times with phosphate-buffered saline (PBS) and subsequently incubated in HiChIP lysis buffer (10 mM Tris–HCl, pH 8.0, 10 mM NaCl, 0.2% NP-40 and freshly added protease inhibitor cocktail) at 4°C for 2 h with rotation. After washing once with cold HiChIP lysis buffer and centrifugation, the cell pellet was resuspended in 0.5% sodium dodecyl sulfate (SDS, 100 μl) and incubated at 62°C for 10 min. SDS was later quenched by adding 25 μl of freshly prepared 10% Triton X-100 and 135 μl water. After incubation at 37°C for 15 min, the resulting chromatin was restriction-digested by adding 25 μl of 10× rCutsmart buffer (NEB) and 100 units of MboI (NEB). Chromatin was digested overnight at 37°C with shaking at 900 rpm. MboI was inactivated by incubation at 62°C for 20 min and then cooling to room temperature. To the mixture were subsequently added 15 nmol each of dCTP, dGTP, dTTP (NEB), biotin-14-dATP (Jena Bioscience) and 40 U Klenow fragment (NEB) in a total volume of 300 μl to perform nucleotide fill-in and biotin labeling. Following incubation at 37°C with shaking at 900 rpm for 1 h, a DNA ligase master mix, which contained 664 μl water, 120 μl of 10× T4 ligase buffer (NEB), 10% Triton X-100, 6 μl of 20 mg/μl bovine serum albumin (BSA) and 10 μl T4 DNA ligase (NEB), was added to the reaction mixture, and the mixture was incubated at room temperature for 6 h. The chromatin was collected by centrifugation and sonicated into 300–500 bp DNA fragments in RIPA buffer (10 mM Tris–HCl, pH 8.0, 140 mM NaCl, 1 mM EDTA, 1% Triton X-100, 0.1% SDS, 0.1% sodium deoxycholate) and then incubated with 10 μg POLR2A antibody (Thermo Fisher Scientific) at 4°C overnight. Antibody-bound chromatin was captured by 50 μl Protein A/G magnetic beads (Thermo Fisher Scientific) pre-blocked with PBS/BSA (5 mg/ml BSA in 1× PBS). The beads were subsequently washed with a low-salt RIPA buffer (10 mM Tris–HCl, pH 8.0, 140 mM NaCl, 1 mM EDTA, 1% Triton X-100, 0.1% SDS, 0.1% sodium deoxycholate) three times, a high-salt RIPA buffer (10 mM Tris–HCl, pH 8.0, 300 mM NaCl, 1 mM EDTA, 1% Triton X-100, 0.1% SDS, 0.1% sodium deoxycholate with proteinase inhibitor cocktail) three times, a LiCl washing buffer (10 mM Tris–HCl, pH 8.0, 150 mM LiCl, 1 mM EDTA, 0.5% NP-40, 0.1% sodium deoxycholate) three times and a TE buffer (10 mM Tris–HCl, pH 8.0, 0.1 mM EDTA) twice. DNA was purified by DNA Clean & Concentrator-5 (Zymo Research), and subsequently quantified using Qubit (Thermo Fisher Scientific). Biotin-labeled DNA was enriched using the Dynabeads MyOne streptavidin C1 (Thermo Fisher Scientific). For each sample, 20 μl streptavidin C1 beads were washed twice with 400 μl Tween wash buffer (5 mM Tris–HCl, pH 7.5, 0.5 mM EDTA, 1 M NaCl, 0.05% Tween 20) and resuspended in 40 μl of 2× binding buffer (10 mM Tris–HCl, pH 7.5, 1.0 mM EDTA, 2.0 M NaCl) and incubated with 40 μl of the above-mentioned DNA isolated from ChIP procedures at room temperature for 15 min. The DNA-bound beads were washed twice with 500 μl Tween wash buffer, collected and resuspended in 25 μl TE buffer. Sequencing libraries were constructed using NEBNext Ultra DNA Library Prep Kit for Illumina (NEB) following the manufacturer’s instructions. The library was paired-end sequenced (2 × 100 bp) on an MGI 2000 platform (BGI). Two biological replicates were performed for each condition.

Paired-end reads were processed using HiC-Pro with default parameter (version 3.1.0) (31). Fastq files were aligned to GRCh38 assembly, deduplicated and assigned to MboI restriction fragments. After filtration for valid interactions, interaction matrices were constructed and visualized using HiCExplorer (32).

### RNA-seq and data analysis

Total RNA was extracted from cells using Omega Total RNA Kit I accordingly to the manufacturer’s recommended procedures. Poly(A) RNA enrichment was conducted using NEBNext Poly(A) mRNA Magnetic Isolation Module (NEB), and the sequencing library was constructed by using the NEBNext Ultra II RNA Library Prep Kit (NEB) following the manufacturer’s instructions. The resulting library was subjected to sequencing analysis on an MGI 2000 platform. The sequencing reads were aligned to GRCh38 assembly using STAR (v.2.7.0) with default parameters (33). Transcript quantification was conducted using featureCounts (v.2.0.3) (34). Differential gene expression analysis was performed with DESeq2 (v.1.36.0) (35).

### Real-time quantitative polymerase chain reaction

Total RNA was extracted using Omega Total RNA Kit I (Omega) following the vendor’s recommended procedures and quantified. Approximately 2 μg total RNA was immediately reverse-transcribed using 200 units of M-MLV reverse transcriptase (Promega) with 1.0 μg oligo(dT)_20_ primer according to manufacturer’s recommended procedures. Real-time quantitative polymerase chain reaction (RT-qPCR) experiments were performed using Luna Universal qPCR Master Mix (NEB) on a CFX96 RT-qPCR detection system (Bio-Rad), by following the manufacturer’s recommended protocol. Standard curves of each gene amplification product were obtained. Correlation coefficients for the standard curves were confirmed to be at least 0.99, and the amplification efficiencies were verified to be within 90–110% (Supplementary Figure S1). Relative quantifications of the genes of interest were conducted based on standard curves and normalized to *GAPDH*. Primers used in RT-qPCR are listed in Supplementary Table S1.

### 3C-qPCR

3C-qPCR was performed as previously described (36) with some modifications. Briefly, 10 million HepG2 cells (mock-treated or treated with 20 μM PDS for 24 h; dimethyl sulfoxide-treated or treated with 1 μM JQ1 for 24 h) were cross-linked in freshly prepared 1% formaldehyde in PBS buffer at room temperature for 10 min and then quenched by incubating with 125 mM glycine at room temperature for 10 min. The cells were harvested, and the cell pellet was suspended in 1 ml cold lysis buffer (10 mM Tris–HCl, pH 7.5, 10 mM NaCl, 0.2% NP-40 with freshly added protease inhibitor cocktail) and incubated with rotation at 4°C for 3 h. After centrifugation at 400 × *g* at 4°C for 5 min, the resulting nuclear pellet was resuspended in 0.5 ml of 1.2× restriction enzyme buffer (60 μl of 10× rCutsmart buffer and 440 μl water) and transferred to a 37°C thermomixer. To the reaction mixture was then added 7.5 μl of 20% (w/v) SDS, and the mixture was incubated at 37°C with shaking at 900 rpm for 1 h. After quenching the SDS with 50 μl of 20% (v/v) Triton X-100 at 37°C for 1 h, the reaction mixture was digested overnight with 400 U EcoRI-HF (NEB) at 37°C with shaking at 900 rpm. Another round of digestion was performed by adding a new aliquot of EcoRI-HF (400 U) to the reaction mixture the next day, and the mixture was incubated at 37°C with shaking at 900 rpm for 2 h. The restriction enzyme was subsequently deactivated by addition of 40 μl of 20% (w/v) SDS and incubation at 65°C for 25 min. The reaction mixture was then diluted with 700 μl of 10× T4 ligase buffer, 5.425 ml ddH_2_O and 375 μl of 20% (w/v) Triton X-100 and incubated at 37°C with gentle shaking for 1 h. To the resulting mixture was added 2000 U T4 ligase (NEB), and the mixture was incubated at 16°C overnight. The sample was then treated with 300 μg proteinase K and the cross-linking was reversed by heating at 65°C overnight. RNA was removed by incubating with 300 μg RNase A at 37°C for 1 h. DNA was purified by phenol–chloroform extraction. Real-time PCR quantifications of ligation products were performed using Luna Universal qPCR Master Mix (NEB) on a CFX96 RT-qPCR detection system (Bio-Rad) following the manufacturer’s recommended protocol. A digested and religated bacterial artificial chromosome (BAC CH17-30P14), covering the genomic regions of interest, was used as a control template. Primers were designed to be in the same direction and as close to the EcoRI restriction sites as possible. A constant primer and a test primer were used in each qPCR reaction. Standard curves of ligation products were constructed using serial dilution of control template (Supplementary Figure S2). The 3C-qPCR data were normalized to a control interaction localized in the *ERCC3* gene. Primers used in 3C-qPCR are listed in Supplementary Table S2.

### ChIP-qPCR

ChIP experiments were conducted as previously described with a few modifications (37). Briefly, 10 million HepG2 cells were cross-linked in freshly prepared 1% formaldehyde in PBS buffer at room temperature for 10 min and then quenched by incubating with 125 mM glycine at room temperature for 10 min. The cells were harvested, and the cell pellet was suspended in 1 ml cold lysis buffer (10 mM Tris–HCl, pH 7.5, 10 mM NaCl, 0.2% NP-40 with freshly added protease inhibitor cocktail) and incubated with rotation at 4°C for 1 h. After centrifugation at 400 × *g* at 4°C for 5 min, the resulting nuclear pellet was resuspended in RIPA buffer (10 mM Tris–HCl, pH 8.0, 140 mM NaCl, 1 mM EDTA, 1% Triton X-100, 0.1% SDS, 0.1% sodium deoxycholate) and incubated with rotation at 4°C for 30 min. Chromatin was sheared using a QSONICA sonicator Q125 at 4°C for 10 min (10 s on/10 s off pulse) with a 42% amplitude. The resulting mixture was centrifuged at 13 200 × *g* at 4°C for 15 min. The supernatant was incubated with 5 μg POLR2A antibody (Thermo Fisher Scientific) at 4°C overnight. Antibody-bound chromatin was captured by 50 μl Protein A/G magnetic beads (Thermo Fisher Scientific). The beads were subsequently washed with a low-salt RIPA buffer (10 mM Tris–HCl, pH 8.0, 140 mM NaCl, 1 mM EDTA, 1% Triton X-100, 0.1% SDS, 0.1% sodium deoxycholate) three times, a high-salt RIPA buffer (10 mM Tris–HCl, pH 8.0, 300 mM NaCl, 1 mM EDTA, 1% Triton X-100, 0.1% SDS, 0.1% sodium deoxycholate with proteinase inhibitor cocktail) once, a LiCl washing buffer (10 mM Tris–HCl, pH 8.0, 150 mM LiCl, 1 mM EDTA, 0.5% NP-40, 0.1% sodium deoxycholate) once and a TE buffer (10 mM Tris–HCl, pH 8.0, 0.1 mM EDTA) once. DNA was purified by DNA Clean & Concentrator-5 (Zymo Research). Quantitative PCR was conducted using Luna Universal qPCR Master Mix (NEB) on a CFX96 RT-qPCR detection system (Bio-Rad) following the manufacturer’s recommended protocol. Primers used in ChIP-qPCR are listed in Supplementary Table S3.

## RESULTS

### Overlapping analysis revealed a correlation between RNAPII-mediated long-range DNA interaction and G4 structures

**Figure 1.**
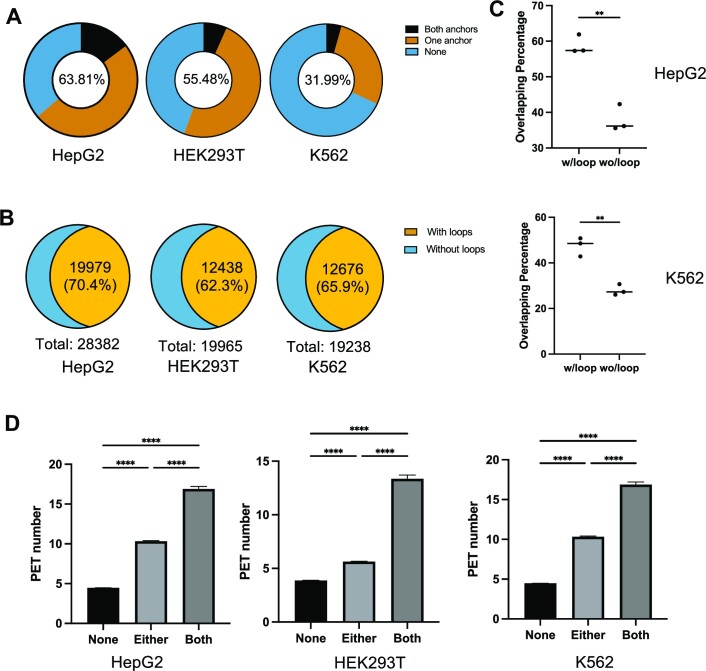
Overlapping analysis between RNAPII ChIA-PET and BG4 ChIP-seq peaks in HepG2, HEK293T and K562 cells. (**A**) Percentages of DNA loop anchors, as revealed from RNAPII ChIA-PET analysis, that overlap with G4 structure loci, as determined from BG4 ChIP-seq analysis. The ChIA-PET loop anchors are divided into three groups, with both anchors having G4 structures (‘Both anchors’), only one of them having G4 structure (‘One anchor’) or neither having G4 structures (‘None’). (**B**) Percentages of G4 structure sites (obtained from BG4 ChIP-seq) that overlap with anchors of RNAPII-mediated DNA loops (obtained from ChIA-PET analysis). (**C**) The percentages of G4 structure peaks overlapping with those RNAPII binding sites that are involved with DNA loop formation versus those that are not. Two-tailed Student’s *t*-test with Welch’s correction: **P < 0.01. (**D**) Statistical analysis of PET number of DNA loops with regard to anchor’s overlapping with BG4 ChIP-seq peaks; shown are mean ± SEM. One-way ANOVA test: ****P < 0.0001.

To investigate the correlation between RNAPII-dependent 3D genome organization and DNA G4, we assessed the co-occupancy of endogenous G4 structure loci with the two anchors of RNAPII-linked DNA loops identified from ChIA-PET analysis (38). To this end, we performed overlapping analysis using POLR2A ChIA-PET data retrieved from the ENCODE database and BG4 ChIP-seq results obtained for the same cell lines, i.e. HepG2, K562 and HEK293T cells (6,14,28,39). Our results showed that large percentages of DNA loops (141 010/220 992, 63.8% in HepG2 cells; 59 729/186 714, 32.0% in K562 cells; and 96 902/174 673, 55.5% in HEK293T cells) carried at least one G4 structure in the two anchors (Figure 1A and Supplementary Table S4). When compared to CTCF ChIA-PET data, we observed a higher overlapping percentage of RNAPII-mediated long-range DNA interactions with endogenous G4 sites, indicating active engagement of DNA G4 structures in transcription (Supplementary Table S5). Additionally, the majority of endogenous G4 sites (19 979/28 382, 70.4% in HepG2 cells; 12 676/19 238, 65.9% in K562 cells; and 12 438/19 965, 62.3% in HEK293T cells) are associated with RNAPII-linked long-range DNA interactions (Figure 1B and Supplementary Table S6), supporting a positive correlation between G4 structure and RNAPII-mediated DNA looping.

We also analyzed the RNAPII-linked DNA loops in HepG2 cells and found that they encompass not only P–E interactions (24.1%), but also P–P interactions (8.8%), E–E interactions (24.4%) and those not involving annotated promoters or enhancers (42.7%) (Supplementary Figure S3). Similar findings were made for those RNAPII-associated DNA loops with at least one anchor containing G4 structure, though with slightly higher percentages P–P (12.7%) and P–E (31.5%) interactions (Supplementary Figure S3).

Because there is a significant enrichment of GC content in promoter region of human genes (40), it is important to examine whether such a positive correlation arises from a bias in sequence context or open chromatin. To explore this possibility, we randomly chose the same number of peaks in control regions that consist of GC-rich sequences and exhibit G4-forming potential *in vitro*, i.e. those regions with observed G-quadruplex sequences (OQS) (41). In both K562 and HepG2 cells, RNAPII-linked DNA loops display a significantly weaker co-occurrence with randomly picked regions with OQS (*P* < 0.01, Monte Carlo simulation) than with those loci enriched with G4 structures. We also conducted a similar analysis for open chromatin as reflected by DNase I hypersensitive sites. The results showed that the RNAPII-linked DNA loops displayed mean overlapping percentages of 35.6% and 11.4% with DNase I hypersensitive sites in HepG2 and K562 cells, respectively, which are substantially lower than with those sites harboring G4 structures (*P* < 0.01, Monte Carlo simulation) (Supplementary Table S7). These results suggest that the enrichment of RNAPII-linked DNA loops at endogenous G4 structure loci is not simply due to the primary sequence of DNA elements at those sites (i.e. being GC-rich) or the association of those loci with open chromatin, but rather attributed to the formation of G4 structures at these sites.

G4s were proposed to be binding hubs for transcription factors to promote active transcription (14). As RNAPII binding sites are directly associated with transcription activity, we assessed the G4 overlapping at those RNAPII binding sites, as revealed by ChIP-seq data, with long-range DNA interactions and those without. Our results showed a much higher G4 percentage at those RNAPII binding loci that are involved with DNA looping (Figure 1C), underscoring the role of G4s in RNAPII-associated long-range DNA interactions.

To further investigate the relationship between DNA looping and G4, we calculated the DNA interaction PET numbers measured by ChIA-PET assay on the basis of G4 overlapping status. The results from HepG2, HEK293T and K562 cells showed significantly higher interaction frequencies when one anchor of DNA loops overlapped with G4 sites in chromatin compared to those loops not associated with cellular G4s. Additionally, more pronounced interactions were detected in cases where both loop anchors carry G4 structures (Figure 1D). These results indicate an active participation of G4s in RNAPII-mediated long-range DNA interactions.

G4 landscapes are distinct in different cells (6), and so are 3D genome organizations (42). Thus, we queried, using Diffbind algorithm (43), differential G4 sites in HepG2 and K562 cells based on statistically significant differences in BG4 ChIP signal (Supplementary Figure S4). A total of 18 991 differential G4 structure sites were retrieved, including 9726 and 9265 in HepG2 and K562 cells, respectively. Likewise, a total of 10 956 cell type-specific DNA loops were called from ChIA-PET datasets for the two cell lines. We next compared the overlapping pattern between differential G4s and long-range DNA interactions (44). Those DNA loops preferentially detected in HepG2 cells are more likely to overlap with G4 structures specifically detected in HepG2 cells than those detected uniquely in K562 cells (4378 versus 506, total 6598); the same finding was made for DNA loops preferentially detected in K562 cells (2347 versus 168, total 4387) (Supplementary Figure S5). These data again suggest the involvement of cellular G4 structures in RNAPII-mediated long-range DNA interactions.

### PDS preferentially diminished RNAPII-mediated long-range DNA interactions involving G4 structure loci

**Figure 2.**
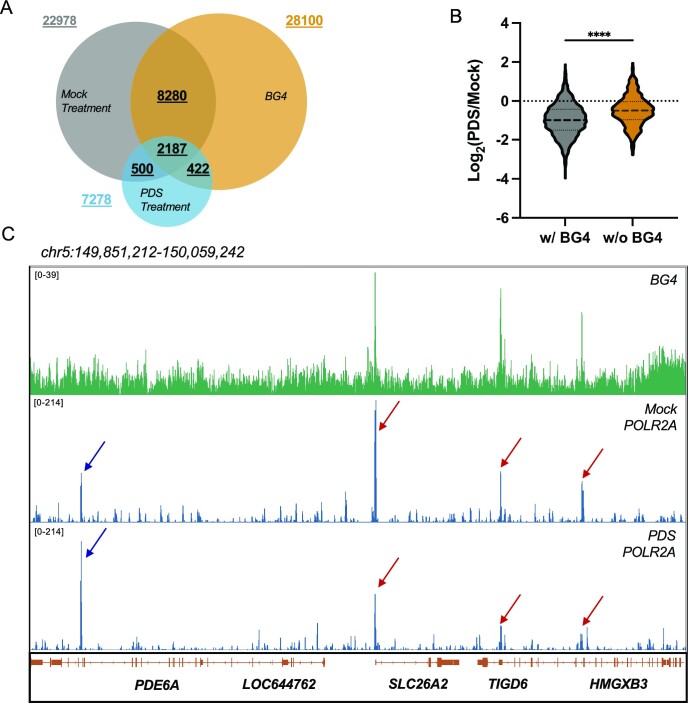
PDS treatment modulates genome-wide landscape of RNAPII occupancy. (**A**) A Venn diagram displaying the overlaps of RNAPII peaks in HepG2 cells that are mock- or PDS-treated, as revealed from HiChIP-seq analysis, with BG4 ChIP-seq peaks detected in HepG2 cells. (**B**) The ratios of RNAPII ChIP-seq signal in PDS-treated over mock-treated HepG2 cells for those peaks that overlap (w/ BG4) or not (w/o BG4) with BG4 ChIP-seq peaks. Two-tailed Student’s *t*-test with Welch’s correction: ****P < 0.0001. (**C**) IGV plots depicting diminished RNAPII ChIP signal at G4 structure loci following PDS treatment.

**Figure 3.**
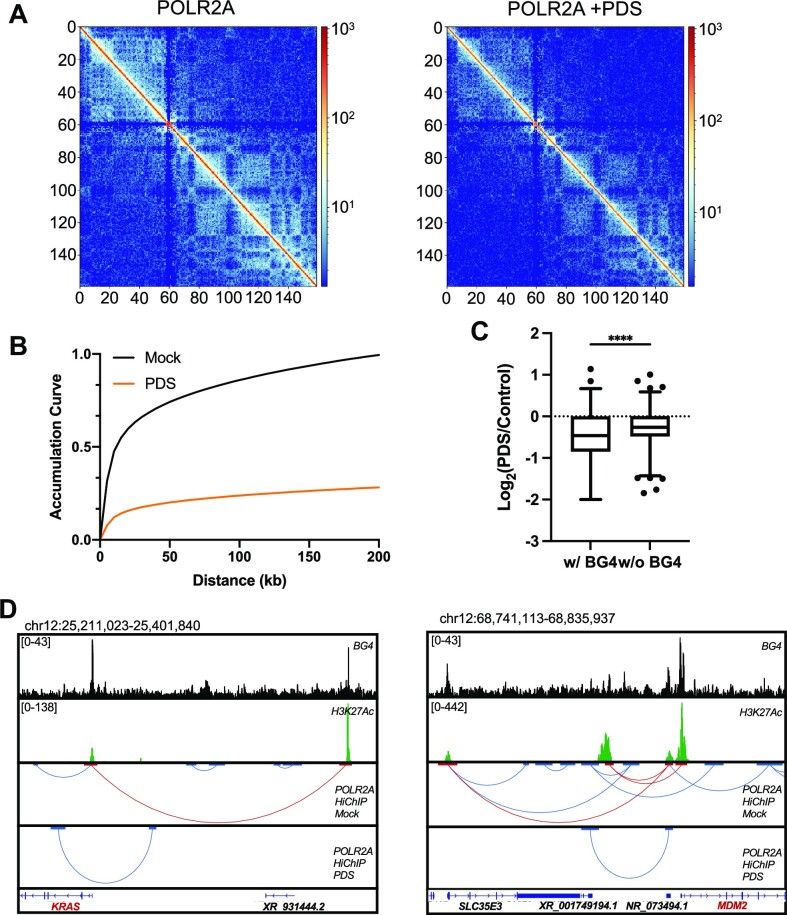
HiChIP-seq analysis showing that PDS preferentially disrupts RNAPII-linked long-range DNA interactions involving G4 structure loci. (**A**) HiChIP interaction matrices of RNAPII in chromosome 7 in HepG2 cells that were mock-treated (left) or treated with PDS (right). (**B**) Aggregation analysis of RNAPII-mediated long-range DNA interactions in mock- and PDS-treated HepG2 cells. (**C**) HiChIP PET ratios in PDS-treated over mock-treated HepG2 cells with respect to overlap with BG4 ChIP-seq peaks. Two-tailed Student’s *t*-test with Welch’s correction: ****P < 0.0001. (**D**) POLR2A HiChIP-seq results for G4-mediated long-range DNA interactions involving the promoters of *KRAS* and *MDM2* genes in mock- and PDS-treated HepG2 cells.

To explore further the origins of the positive correlation between G4 structures and RNAPII-mediated DNA looping, we conducted POLR2A HiChIP-seq in HepG2 cells with or without PDS treatment. In this context, PDS, a small-molecule G4 ligand that binds specifically to G4 structures, has been widely used for displacing G4-binding proteins from G4 sites in cells (13,45,46). As HiChIP-seq also captures the *in vivo* binding landscape of target proteins, we first examined the effect of PDS on RNAPII binding profiles. A total of 22 978 RNAPII peaks were identified in ‘mock’ condition and exhibited a high overlapping percentage (10 467/22 978, 45.5%) with BG4 ChIP-seq peaks. Following a 24-h treatment with 20 μM PDS, only 7278 RNAPII binding sites were captured and overlapping analysis showed an attenuated co-occurrence of the RNAPII binding loci with endogenous G4 structures (2609/7278, 35.8%) (Figure 2A). Likewise, signal intensities of RNAPII are strongly diminished in cells treated with PDS than those without (mock), where the signal ratio of PDS/mock for G4 loci was significantly lower than non-G4 loci (Figure 2B), indicating a role of PDS in impairing the recruitment of RNAPII to G4 structure sites in cells. Integrative Genomics Viewer (IGV) plots of representative regions showed a strong effect of PDS on displacing RNAPII from the promoters of *SLC26A2*, *TIGD6* and *HMGXB3* genes that carry G4 structures (Figure 2C). However, RNAPII ChIP signal was augmented in non-G4 regions after PDS treatment, which is consistent with the above-mentioned statistical analysis. In this vein, our cell viability assay results showed that a 24-h exposure with 20 μM did not give rise to any apparent alteration in the viability of HepG2 cells (Supplementary Figure S6). We next examined the impact of G4 structures on RNAPII-mediated long-range DNA interactions. The results from HiChIP-seq analysis showed that PDS treatment markedly attenuated RNAPII-mediated DNA looping, as shown in the chromosome matrix view (Figure 3A). In addition, a pronouncedly decreased number of DNA loops were detected in PDS-treated cells relative to mock-treated cells (66 417 versus 18 778), supporting that G4 is crucial for RNAPII-linked long-range DNA contacts. We also observed an attenuated presence of G4 structure sites in the loop anchors of the detected long-range DNA interactions in PDS-treated relative to mock-treated HepG2 cells (27.7% versus 33.8%, Supplementary Table S7). Moreover, those DNA loops not perturbed by PDS treatment exhibited a much lower extent of overlap with endogenous G4 loci (Supplementary Table S8), indicating that PDS preferentially disrupts G4-mediated long-range DNA interactions.

Genome-wide accumulation analysis of RNAPII-associated DNA loops in a distance range of 5–200 kb showed much weaker RNAPII-linked DNA interactions following PDS treatment (Figure 3B). Furthermore, those RNAPII-mediated DNA loops overlapped with endogenous G4 loci displayed a more pronounced diminution in long-range interaction frequency in cells treated with PDS than those lacking overlap with endogenous G4 sites (Figure 3C).

Previous studies showed the enrichment of G4 structures at the promoters of oncogenes, including *KRAS* and *MDM2* (47,48). Thus, we examined whether G4 structures play any role in RNAPII-mediated DNA interactions of those oncogenes. Analysis of our HiChIP-seq data revealed multiple G4-containing long-range DNA interactions involving the promoters of *KRAS* and *MDM2* genes, and the disruption of these interactions following PDS treatment (Figure 3D). In this vein, our RNAPII ChIP-qPCR experiment revealed enrichments of RNAPII at both the promoters and enhancers of *KRAS* and *MDM2* genes (Supplementary Figure S7), which are also enriched with G4 structures (Figure 3D).

Together, these data furnish evidence to support that RNAPII-mediated long-range DNA interactions are highly associated with G4 structures, and G4-binding ligand could perturb RNAPII-linked and G4-dependent DNA loops, including those involving promoters of oncogenes.

### G4-dependent DNA loops regulate gene expression

RNAPII is responsible for mRNA transcription and plays a vital role in gene expression (49). In light of the above results showing that cellular G4s are highly correlated with RNAPII-mediated DNA loops, we next asked whether G4-dependent long-range DNA interactions modulate gene expression.

**Figure 4.**
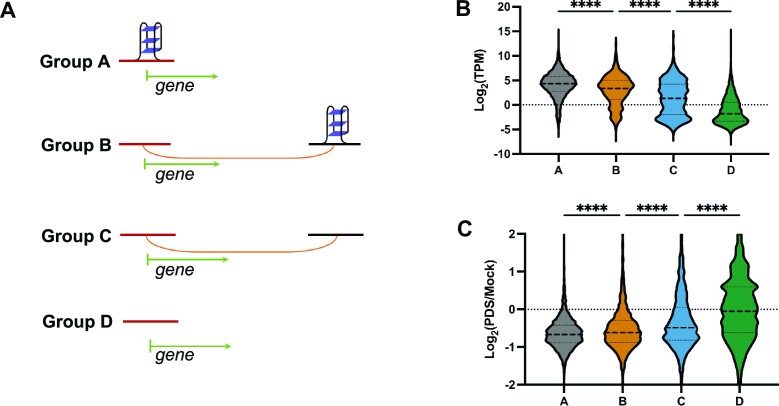
Consolidation analysis of RNA-seq and 3D genome architecture mapping. (**A**) A scheme depicting the grouping strategy. Genes were divided into four groups based on their association with G4 structures: group A genes have G4 structures in their promoters; group B and C genes do not contain G4 structures in their promoters, but these promoters are connected, via RNAPII-mediated NDA looping, to distal sites with and without G4 structures, respectively. The remaining genes were classified into group D. (**B**) Transcriptome profiles of each group of genes in mock-treated HepG2 cells. (**C**) Statistical analysis of PDS-induced alterations of the transcriptome in the four groups of genes in HepG2 cells. One-way ANOVA test: ****P < 0.0001.

As G4 structures are proposed to be correlated with active transcription (5,11,14,19), we first evaluated the expression pattern of genes with respect to the presence of G4 structures (based on BG4 ChIP-seq data) and long-range DNA interactions (based on ChIA-PET data). We divided genes into four groups based on their associations with G4 structures (Figure 4A): genes in group A carry G4 structures in their promoters; group B and C genes do not contain G4 structures in their promoters, but form loops with a distal site with (group B) or without (group C) G4 structure; and the remaining genes were classified into group D. Among these four groups of genes, group A exhibits the highest expression level, and group B displays significantly higher expression profile than group C. Those genes without any RNAPII-linked long-range interactions or associated with G4 structures (group D) exhibit the lowest expression profile (Figure 4B).

Next, we evaluated the influence of PDS treatment on cellular transcriptome. We found that genes in the aforementioned groups A and B exhibited diminished expression after PDS treatment (Supplementary Figure S8); such diminished expression, however, is much less pronounced for group C genes, and not observed for those in group D (Figure 4C). These data underscored that G4 structures not only locally modulate expression of target genes through their promoters, but also distally regulate the transcription of target genes through RNAPII-linked long-range DNA interactions.

### G4-dependent DNA loops activate the expression of *AKR1C* family genes

**Figure 5.**
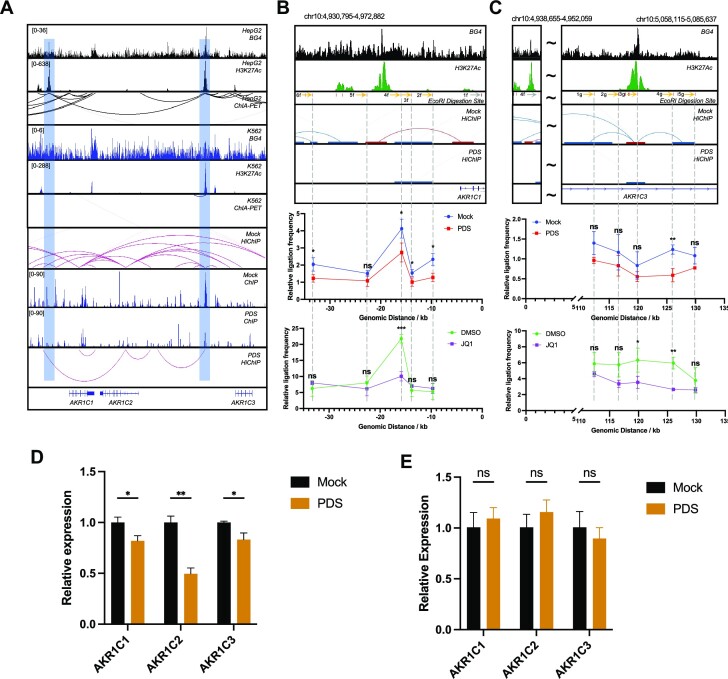
G4-dependent and RNAPII-linked DNA loops regulate the expression of *AKR1C* family genes. (**A**) RNAPII-mediated long-range DNA interactions within the regions of *AKR1C1–3* genes in HepG2, but not in K562 cells. POLR2A HiChIP-seq results for the regions of *AKR1C1–3* genes in mock- and PDS-treated HepG2 cells. (**B**, **C**) 3C-qPCR results for *AKR1C1* E–P interactions and G4-mediated E–E interactions in HepG2 cells with or without PDS treatment, and with or without JQ1 treatment. The relative level of each ligation product was plotted according to its distance from the constant primer. The data were normalized to *ERCC3* control interaction frequencies for each ligation product. The data represent mean ± SD (*n*= 3). Two-tailed Student’s *t*-test: *, 0.01 ≤ *P* < 0.05; **, 0.001 ≤ *P* < 0.01; ***, 0.0001 ≤ *P* < 0.001. (**D**, **E**) RT-qPCR (mean ± SD, *n*= 3) results showing the relative expression levels of *AKR1C1–3* genes in HepG2 and K562 cells with or without PDS treatment. Two-tailed Student’s *t*-test with Benjamini and Hochberg correction for multiple comparison: *, 0.01 ≤ *P* < 0.05; **, 0.001 ≤ *P* < 0.01.

The *AKR1C1–3* genes are closely located on chromosome 10 in a region spanning ∼200 kb. RNAPII ChIA-PET data of HepG2 cells revealed multiple DNA loops within this region and two G4 structures marked with enhancer activity (H3K27Ac) residing in the center of the DNA interaction network (Figure 5A). In contrast, G4 structures are depleted in these regions in K562 cells, which are accompanied with much less RNAPII-mediated DNA interaction network in these regions in K562 cells than HepG2 cells (Figure 5A and Supplementary Figure S9). Such analysis suggests that G4s may play a critical role in 3D genome architecture and modulate the expression of *AKR1C1–3* genes through long-range DNA interactions.

After PDS treatment, RNAPII exhibited markedly diminished occupancy at the two G4 regions, which is accompanied with reduced RNAPII-mediated DNA interactions, as shown in the HiChIP-seq results (Figure 5A and Supplementary Figure S10). To further validate these findings, we conducted 3C-qPCR assay, which can accurately determine interaction frequencies between genomic loci. One interaction of interest is between the promoter of *AKR1C1* and its upstream enhancer (E–P interaction, Figure 5B). We measured ligation efficiencies between the constant fragment (with primer 1f) located in the promoter and five candidate fragments (with primers 2f–6f). Our results showed a markedly elevated ligation efficiency between 1f and 4f, which represents the physical interaction of *AKR1C1* promoter and upstream G4-containing enhancer in HepG2 cells under mock treatment conditions. In accordance with HiChIP-seq results, we observed a significant decrease in ligation efficiency after PDS treatment, supporting an active role of G4 structure in this E–P interaction. Previous studies demonstrated that JQ1, a small molecule, can specifically dislodge BRD4 from enhancers, thereby dissolving mediator and RNAPII clusters (50,51). Treatment with JQ1 can cause reconfiguration of chromatin structure in selected gene loci (52). We observed a significantly diminished ligation efficiency in the same region following JQ1 treatment, indicating that the interaction observed between the transcription start site region of *AKR1C1* and the distal G4 is P–E interaction.

We also validated another interaction between two G4 structures in *AKR1C1–3* region. These two G4 structures serve as hubs in connecting multiple genomic loci and overlap with H3K27Ac enhancer marks. Because of the long distance between these two regions (∼120 kb), a lower ligation frequency was observed compared to the aforementioned E–P interaction (Figure 5C). However, 3C-qPCR assay showed that PDS treatment resulted in a significant attenuation in interaction between the two G4 loci (4f and 4g) compared to mock treatment (Figure 5C). Such diminution was also observed in cells treated with JQ1 (Figure 5C). These results demonstrated a physical linkage between two G4s involved in an E–E interaction.

Next, we examined the role of G4-dependent RNAPII-mediated DNA loops in the expression of *AKR1C1–3* genes. In accordance with the diminished RNAPII-mediated DNA interactions in this region, we observed attenuated expressions of *AKR1C1–3* genes in HepG2 cells (Figure 5D). In contrast, we did not observe any significant changes in expression of *AKR1C1–3* genes in K562 cells after PDS treatment, which is consistent with the lack of G4 structure and DNA loops in these regions in K562 cells (Figure 5E). The above data support a role of G4 structures in augmenting the occupancy of RNAPII in enhancer regions to stimulate transcription of target genes brought to close proximity by DNA looping. These results further substantiate the roles of G4 structures in cell type-specific RNAPII-mediated DNA looping and transcription regulation.

## DISCUSSION

Under physiological conditions, G-rich regions of DNA can fold into G4 structures, which regulate important cellular processes, including transcription. DNA G4 was first found to be involved in gene regulation by Hurley and coworkers (53), who observed that treatment of Burkitt’s lymphoma cells with G4 ligands, e.g. PDS and TMPyP4, led to diminished transcription of *MYC* gene, whose promoter contains G4-forming sequence. With the availability of a G4 structure-specific antibody (i.e. BG4), recent studies unveiled an association between G4 and transcription regulation (12,14,54–56). For instance, overlapping analysis of DNMT1 ChIP-seq revealed its significant enrichment at cellular G4 sites, which also exhibit much lower CpG methylation (19). A sequestration model, where the recruitment of DNMT1 to G4 inhibits its enzymatic activity and results in hypomethylated regions, was proposed to account for the role of G4 in modulating gene expression. A later study showed that different G4 folding states, measured by G4 ChIP-seq, are associated with distinct transcriptome profiles in two cell lines (54). Therefore, it is of interest to investigate the detailed mechanism through which DNA G4 structures modulate transcription.

Aside from promoters, transcription can also be modulated by distal regulatory elements such as enhancers, which are remote from transcription start sites of target genes in the primary sequence, but close in 3D genome organization. Mediated by transcription factors and cofactors, E–P interactions initiate and promote RNAPII-mediated transcription (21). Integrative analysis showed significant enrichment of G4 at the TAD boundaries, which are proposed to be the structural scaffolds for E–P contacts. A recent study by Li *et al.* (13) revealed the ability of YY1, a transcription factor known to enable DNA looping (27), in binding with G4 structures *in vitro* and in cells, and found that disruption of YY1–G4 binding led to a diminution in YY1-mediated DNA looping.

With the encouraging results of YY1 transcription factor, we sought to investigate further how G4 functions in RNAPII-linked long-range DNA interactions and affects transcription in general. First, we employed bioinformatic analysis by comparing POLR2A ChIA-PET and BG4 ChIP-seq in three cell lines (i.e. HepG2, HEK293T and K562). We found a strong overlap, >60% in HepG2 cells, between G4 structure sites and RNAPII-mediated DNA loops (Figure 1A). Our finding is consistent with previous integrative analysis of G4 ChIP-seq with the Hi-C dataset (26). Moreover, we analyzed the association between interaction frequency and the presence of G4 structure, and more interactions were detected at cellular G4 loci (Figure 1D). Shuffling calculation at DNase I hypersensitive sites and sites with OQS substantiated our finding that G4 structures are important determinants for long-range DNA contacts (Supplementary Table S7). As DNA loops vary in different cell lines, we demonstrated that distinct DNA looping patterns are strongly associated with the cell type-specific distributions of G4 structures in chromatin (Supplementary Figure S5). Together, our bioinformatic analysis lent evidence to support that G4 is involved in high-order chromatin organization and in RNAPII-mediated long-range DNA interactions. It is worth noting that there is so far no evidence supporting that RNAPII can bind directly with DNA G4 structures. However, many transcription factors exhibit ability in binding directly with G4 DNA (13,16,57–59). As noted above, one of these transcription factors, YY1, could bind to G4 DNA at low nM binding affinity, and this binding contributes to YY1-mediated DNA looping (13). It will be important to examine how other G4-binding transcription factors contribute to RNAPII-mediated DNA looping.

We also found that PDS, a small-molecule G4-binding ligand, could disrupt global RNAPII binding with an ∼68% decrease in significant binding sites (Figure 2A). Specifically, those RNAPII binding loci with G4 structures displayed more pronounced decreases following PDS treatment compared to those without (Figure 2B and C). As RNAPII constitutes the core component of the mammalian transcription machinery, our data suggest an important role of G4 in RNAPII-mediated long-range DNA interaction and transcription regulation. Importantly, by using HiChIP-seq, we detected attenuated RNAPII-mediated long-range DNA interactions following PDS treatment (Figure 3A and B), and we demonstrated that such diminishing effect was more pronounced in DNA loops with G4-containing anchors than those without (Figure 3C).

G4 structure has been proposed to play important roles in transcription regulation (5,11,19); however, limited studies demonstrated experimentally how distal G4s modulate gene expression (13). We combined RNA-seq with long-range DNA interaction data (ChIA-PET/HiChIP-seq) to unravel the regulatory roles of G4-depedent DNA loops in transcription. Transcriptome abundance profiling revealed higher expression of not only those genes with G4 in promoter regions but also those connected to a distal G4 through RNAPII-mediated long-range DNA interactions (Figure 4B). In addition, PDS-induced alterations in expression of those genes with G4-dependent DNA loops are more pronounced than those genes connected with DNA loops lacking G4 structures (Figure 4C).

We further evaluated how G4-dependent DNA loops modulate gene expression in a specific genomic region. AKR1C proteins are a group of enzymes responsible for steroid reductions (60). AKR1C3 was shown to have an important role in the progression of prostate cancer (61) and several selective inhibitors of AKR1C3 have shown antitumor activity (62–64). Furthermore, a bioinformatic analysis showed elevated *AKR1C1–3* expression in liver cancer samples compared with normal liver samples (65). Poorer survival rate was observed in those cancer patients with high expression of *AKR1C1–3*, suggesting that they may serve as prognostic markers for liver cancer (65). Our HiChIP experiments demonstrated that treatment with a G4-binding ligand led to diminished RNAPII-mediated DNA loops (Figure 5A). By using 3C-qPCR assay, we validated our findings made from RNAPII HiChIP-seq and demonstrated the participation of G4 structure in both the P–E and E–E interactions in *AKR1C1–3* regions (Figure 5B and C). We further demonstrated that G4-dependent RNAPII-mediated DNA loops play an important role in regulating the expression of *AKR1C1–3* in HepG2 cells (Figure 5D and E). Considering the possible role of *AKR1C* family in liver cancer, our study in HepG2 cells provided an important knowledge basis for potential therapeutic interventions of liver cancer. In addition, we failed to observe any apparent impact of PDS treatment on the expression of the *AKR1C1–3* genes in K562 cells, which is in line with the lack of G4 structure-mediated DNA looping at these genetic loci in K562 cells. Our work, hence, also underscores a role of the interplay of G4 structure and RNAPII-mediated DNA looping in cell type-dependent gene expression in human cells.

**Figure 6.**
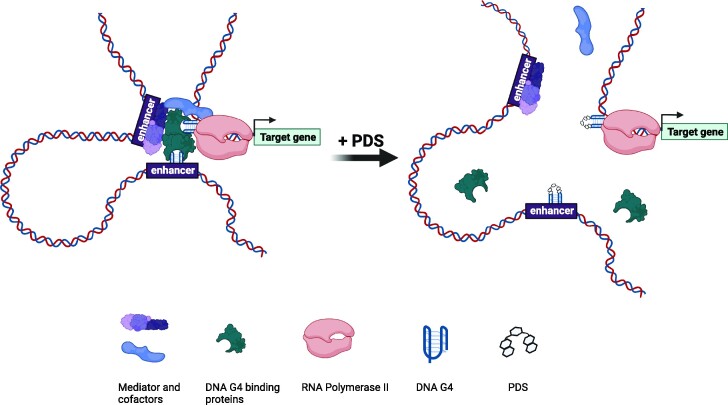
A model illustrating the involvement of G4 structures in RNAPII-linked long-range DNA interactions and in gene expression regulation. A small-molecule G4 ligand, PDS, can perturb G4-binding capacity of proteins (e.g. YY1) and disrupt 3D genome architecture.

In summary, we revealed, using a combination of bioinformatic and experimental approaches, that DNA G4 actively participates in RNAPII-mediated long-range DNA interactions (Figure 6). We also found that G4 structures not only locally modulate transcription in promoter regions, but also remotely regulate gene expression through long-range DNA interactions. Moreover, our work revealed a role of G4 structure in differentially modulating RNAPII-mediated DNA looping and expression of target genes in two different cell lines, which could stimulate future studies about the role of G4-dependent DNA loops in cell type-specific gene expression and in cancer biology. Together, our study provided new insights into the functional interplay of G4 structures and 3D genome architecture in regulating gene expression.

## Supplementary Material

gkad588_Supplemental_File

## Data Availability

The HiChIP-seq and RNA-seq data generated in this study have been deposited into the National Center for Biotechnology Information’s Gene Expression Omnibus database (HiChIP-seq: GSE222206; RNA-seq: GSE222207).
