# Supplementary material for: G-quadruplex DNA contributes to RNA polymerase II-mediated 3D chromatin architecture
Source: Nucleic Acids Res. 2023 Jul 10;51(16):8434–46. doi: 10.1093/nar/gkad588 (PMC10484665; doi:10.1093/nar/gkad588)

Supplementary Materials for

"G-quadruplex DNA Contributes to RNA Polymerase II-mediated 3D Chromatin Architecture"

Jun Yuan[1], Xiaomei He[2], and Yinsheng Wang[1,2]*

[1]Environmental Toxicology Graduate Program and [2]Department of Chemistry, University of California, Riverside, Riverside, CA 92521-0403, United States

*To whom correspondence should be addressed: yinsheng.wang@ucr.edu

Supplementary Table S1. Primers used in RT-qPCR.

**Sequence Names**	**Sequences**
AKR1C1-F	5’-TCCGCCATATTGATTCTGCTC-3’
AKR1C1-R	5’-TGGTCGATGGGAATTGCAC-3’
AKR1C2-F	5’-TGATGGTCACTTCATGCCTG-3’
AKR1C2-R	5’-GCTTCTATTGCCAATTTGACGG-3’
AKR1C3-F	5’-GGGTTCCGCCATATAGATTCTG-3’
AKR1C3-R	5’-ACTCTGGTCGATGAAAAGTGG-3’
TUBB-F	5’-CGGGCAGTGTTTGTAGACTTGG-3’
TUBB-R	5’-CTCCTTGCCAATGGTGTAGTGC-3’

Supplementary Table S2. Primers used in 3C-qPCR.

Sequence Names	Sequences
3C_AKR1C1_1f	5'-CTACAGACCTTCATGGGAAAC-3'
3C_AKR1C1_2f	5'-CCCATCTCCCTTTCCTCATATTC-3'
3C_AKR1C1_3f	5'-TGTGGCCTGAAACACCATAAT-3'
3C_AKR1C1_4f	5'-GAGCCACGTTGGAGCTATT-3'
3C_AKR1C1_5f	5'-CCAAAGAGATTATCTGACACTGGA-3'
3C_AKR1C1_6f	5'-CGCATCACAAACAGCGTATT-3'
3C_AKR1C1_6r (digestion efficiency)	5'-AGGTAGAAATGGGTGCTTCC-3'
3C_AKR1C3_1g	5'-CGTGAGATTGCAACCATTCAG-3'
3C_AKR1C3_2g	5'-GCCATCTGATCCATCCAAA-3'
3C_AKR1C3_3g	5'-GTTAAAGAGCCAGCCTGAGAA-3'
3C_AKR1C3_4g	5'-GTTCCTTGAGGTTGAAGCAAATC-3'
3C_AKR1C3_5g	5'-ATCCTGATTCTGCCTCCTTTC-3'
3C_ERCC3_1f	5'-CACAGAAGGAGGCACTCG-3'
3C_ERCC3_2f	5'-ACAACATCAACTCGTACCTGAA-3'
3C_GAPDH_f	5'- CCTAGGGCTGCTCACATATTC-3'
3C_GAPDH_r	5'- CGCCCAATACGACCAAATCTA-3'

Supplementary Table S3. Primers used in ChIP-qPCR.

**Sequence Names**	**Sequences**
KRAS_promoter_f	5’-AGCCGCCAATTCTGACC-3’
KRAS_promoter_r	5’-GTGCTCTTCGCAGCTTCT-3’
KRAS_enhancer_f	5’-AGACTCCCAAGGGCTCTTTA-3’
KRAS_enhancer_r	5’-GCAATCGGCCTATGCAAATAAG-3’
MDM2_promoter_f	5’-TTTCCCAGCTGTGTTCAGTG-3’
MDM2_promoter_r	5’-TACAAGCAAGTCGGTGCTTAC-3’
MDM2_enhancer_f	5’-TGTCTAGCAAATCGGGTCAAT-3’
MDM2_enhancer_r	5’-TTGCGTCTTTGGGTCCTATC-3’
Negative Site_f	5’-GTGCCATTAGTGACCCTCTTT-3’
Negative Site_r	5’-TGACCCTTTAGTTGACCCTTTATT-3’

Supplementary Table S4. Overlapping of RNAPII-mediated DNA loops with G4 structures *in vivo.* Shown are the numbers of loops (from POLR2A ChIA-PET analysis) with both anchors (Both), one of the two anchors (Either), or neither anchors (None) containing G4 structures, as obtained from BG4 ChIP-seq analysis.

ENCODE Accession #	Target Protein	Cell line	Total	Both	Either	None	Overlapping Percentage
ENCFF030PMM	POLR2A	K562	186714	8199	51530	126985	31.99%
ENCFF759YBZ	POLR2A	K562	115184	4111	25030	86043	25.30%
ENCFF115TNW	POLR2A	HepG2	223490	28910	94082	100498	55.03%
ENCFF364UNM	POLR2A	HepG2	220992	32563	108447	79982	63.81%
ENCFF816HOS	POLR2A	HEK293T	272703	13807	114015	144881	46.87%
ENCFF125JGW	POLR2A	HEK293T	174673	11656	85246	77771	55.47%

Supplementary Table S5. Overlapping of CTCF-mediated loops with G4 structures *in vivo.* Shown are the numbers of loops (from CTCF ChIA-PET analysis) with both anchors (Both), one of the two anchors (Either), or neither anchor (None) containing G4 structures, as obtained from BG4 ChIP-seq analysis.

ENCODE Accession #	Target Protein	Cell line	Total	Both	Either	None	Overlapping Percentage
ENCFF607PZX	CTCF	K562	100265	7653	7646	84966	15.3%
ENCFF118PBQ	CTCF	K562	129670	9032	8949	111689	13.9%
ENCFF299NHM	CTCF	HepG2	48416	8430	8332	31654	34.6%
ENCFF743ZWY	CTCF	HepG2	151731	11931	11904	127896	15.7%
ENCFF158MHM	CTCF	HEK293T	404977	3298	43637	358042	11.6%
ENCFF653GUB	CTCF	HEK293T	326724	3642	44994	278088	14.9%

Supplementary Table S6. Overlapping of G4 structure with RNAPII-mediated loop anchors. Shown are the numbers of BG4 ChIP-seq peaks that overlap with at least one anchor of RNAPII-linked DNA loops.

ENCODE Accession #	Target	Cell line	Total	Overlap with RNAPII loops	Overlapping Percentage
ENCFF030PMM	G4	K562	19238	12676	65.89%
ENCFF759YBZ	G4	K562	19238	9741	50.63%
ENCFF115TNW	G4	HepG2	28382	19740	69.55%
ENCFF364UNM	G4	HepG2	28382	19979	70.39%
ENCFF125JGW	G4	HEK293T	19965	12438	62.29%
ENCFF816HOS	G4	HEK293T	19965	13289	66.56%

Supplementary Table S7. Overlapping of RNAPII-mediated loops with shuffled regions in DHS (DNaseI hypersensitive sites, obtained from ENCODE with accession number ENCFF897NME, ENCFF285OXK and ENCFF274YGF) or OQS (Observed G-quadruplex Sequence, obtained from GEO database with accession number GSE63874, lifted to GRCh38 assembly) background in one representative Monte Carlo simulation. Shown are the numbers of loops (from POLR2A ChIA-PET analysis) with both anchors (Both), one of the two anchors (Either), or neither anchors (None) overlapping with shuffled regions with DHS or OQS.

ENCODE Accession #	Shuffle background	Cell line	Total	Either	Neither	Overlapping Percentage
ENCFF030PMM	DHS	K562	186714	19157	167557	11.4%
ENCFF030PMM	OQS	K562	186714	10198	176516	5.5%
ENCFF364UNM	DHS	HepG2	220992	77952	143040	35.2%
ENCFF364UNM	OQS	HepG2	220992	25244	195748	11.4%
ENCFF125JGW	DHS	HEK293T	174673	55353	119320	31.7%
ENCFF125JGW	OQS	HEK293T	174673	16950	157723	9.7%

Supplementary Table S8. Total numbers of PET connections detected by POLR2A HiChIP and those with at least one anchor overlapping with BG4 ChIP-seq peaks in mock- and PDS-treated HepG2 cells. Similar analysis was performed for those PET connections that are commonly observed in mock- and PDS-treated HepG2 cells (Mock & PDS overlapped).

Condition	PET (≥3)	Overlapped with BG4	Percentage
Mock treatment	66417	22438	33.8%
PDS treatment	18778	5205	27.7%
Mock & PDS overlapped	7333	1110	15.1%

Figure S1. Standard curves of RT-qPCR showing high correlation coefficients and acceptable amplification efficiencies.

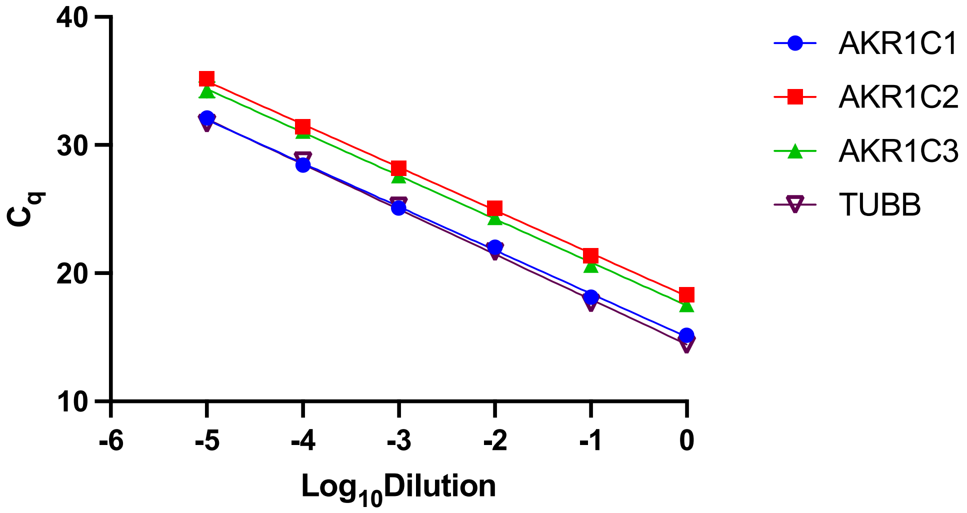

Gene name	Correlation coefficient	Slope	Amplification efficiency
**AKR1C1**	**0.9982**	**-3.392**	**97.2%**
**AKR1C2**	**0.9985**	**-3.355**	**98.6%**
**AKR1C3**	**0.9978**	**-3.378**	**97.7%**
**TUBB**	**0.9978**	**-3.517**	**92.5%**

Figure S2. Standard curves of 3C-qPCR showing high correlation coefficients and acceptable amplification efficiencies.

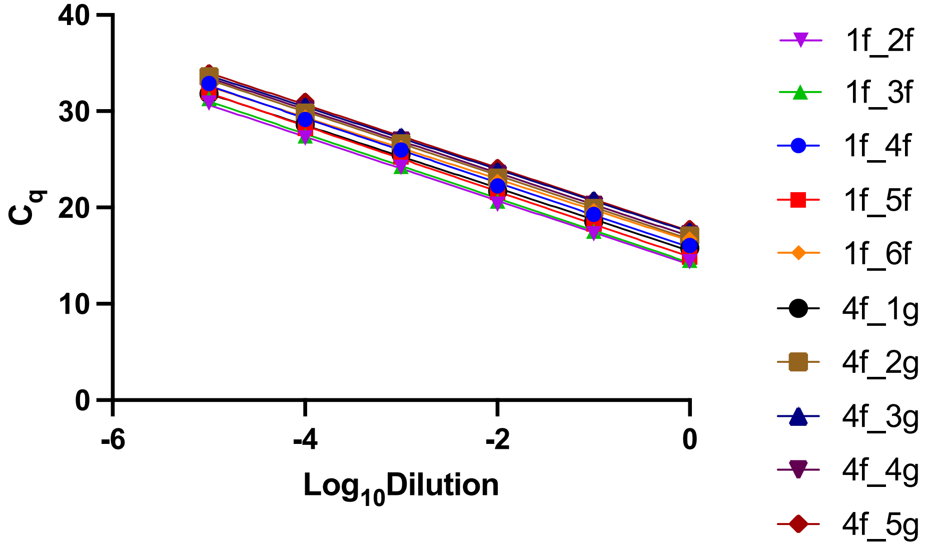

Ligation product	Correlation coefficient	Slope	Amplification efficiency
1f_2f	0.9973	-3.312	100.4%
1f_3f	0.9974	-3.350	98.8%
1f_4f	0.9984	-3.355	98.6%
1f_5f	0.9979	-3.403	96.7%
1f_6f	0.9986	-3.203	105.2%
4f_1g	0.9962	-3.264	102.5%
4f_2g	0.9975	-3.310	100.5%
4f_3g	0.9989	-3.252	103.0%
4f_4g	0.9986	-3.294	101.2%
4f_5g	0.9985	-3.284	101.6%

Figure S3. Annotation of POLR2A ChIA-PET in HepG2 cells regarding to G4 presence.

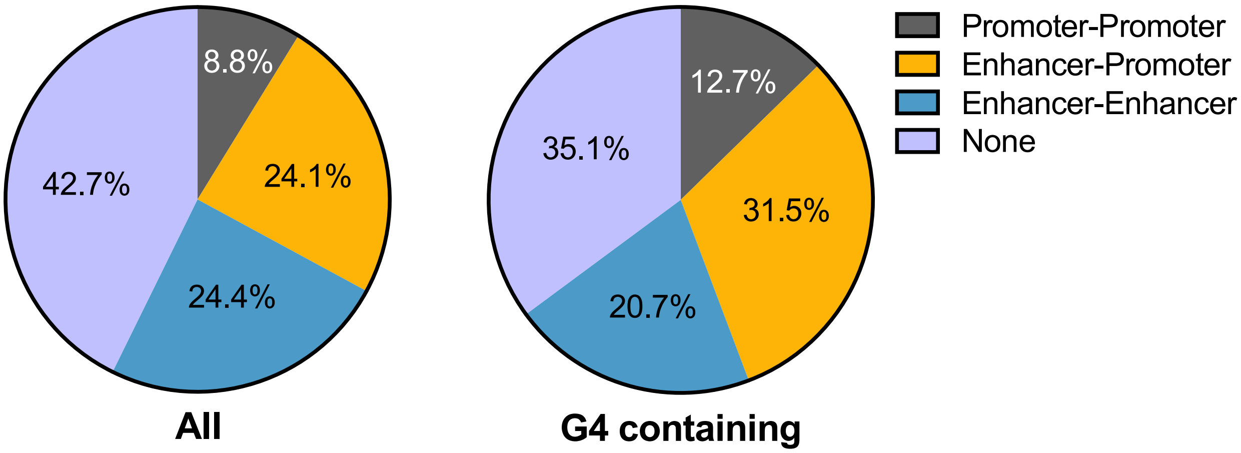

Figure S4. Differential analysis of BG4 ChIP-seq in K562 and HepG2 cells showing a total of 18,991 differential G4 sites (*HepG2-enriched*: red, *K562-enriched*: blue).

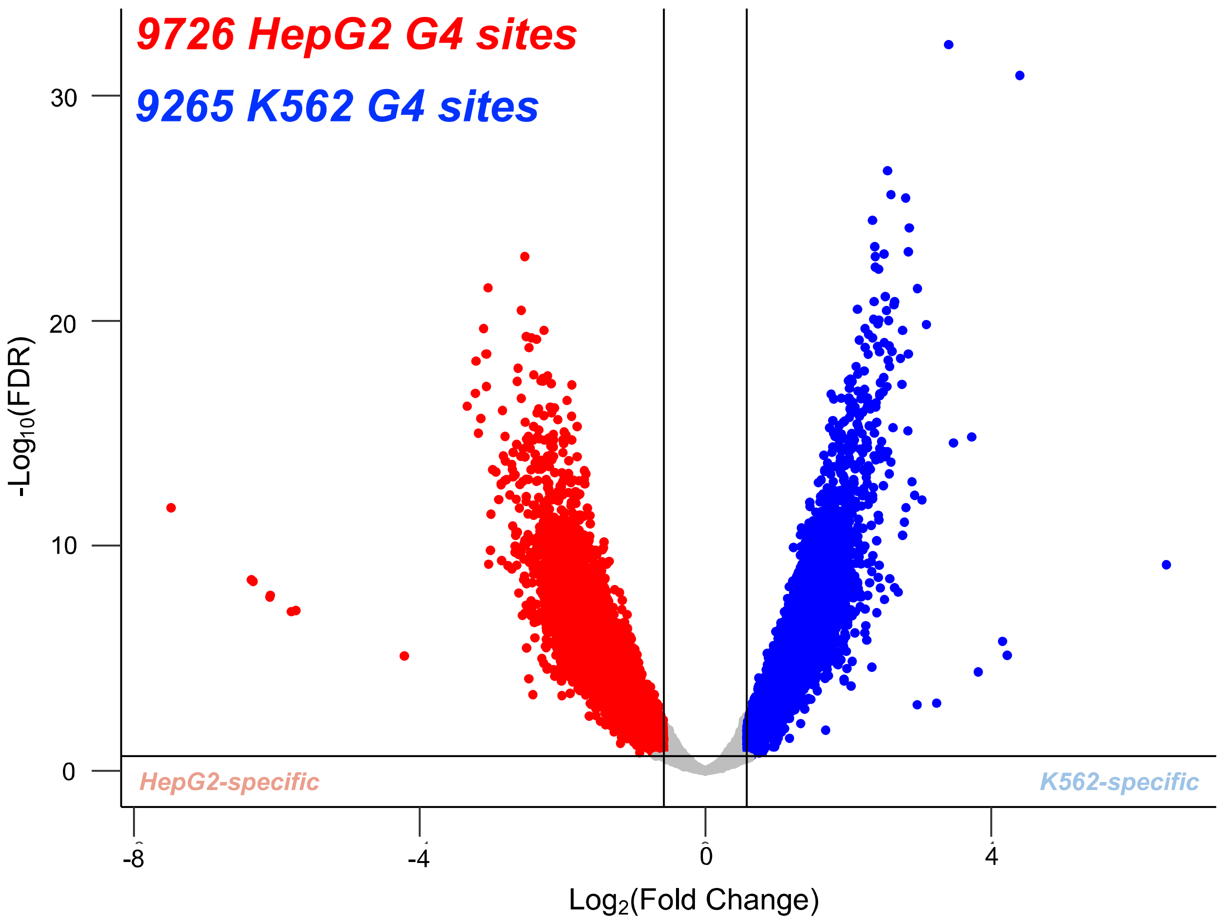

Figure S5. Differential analysis of POLR2A ChIA-PET and BG4 ChIP-seq in K562 and HepG2 cells showing a high level of correlation between G4 structure and RNAPII-mediated long-range DNA interactions. Each dot represents one DNA loop (*HepG2-enriched*: upper-left, *K562-enriched*: upper-right). Color of the dots corresponds to overlapping profile with cell-type specific G4 structures (K562 enriched G4: blue, HepG2 enriched G4: red, No selectivity/overlapping: black).

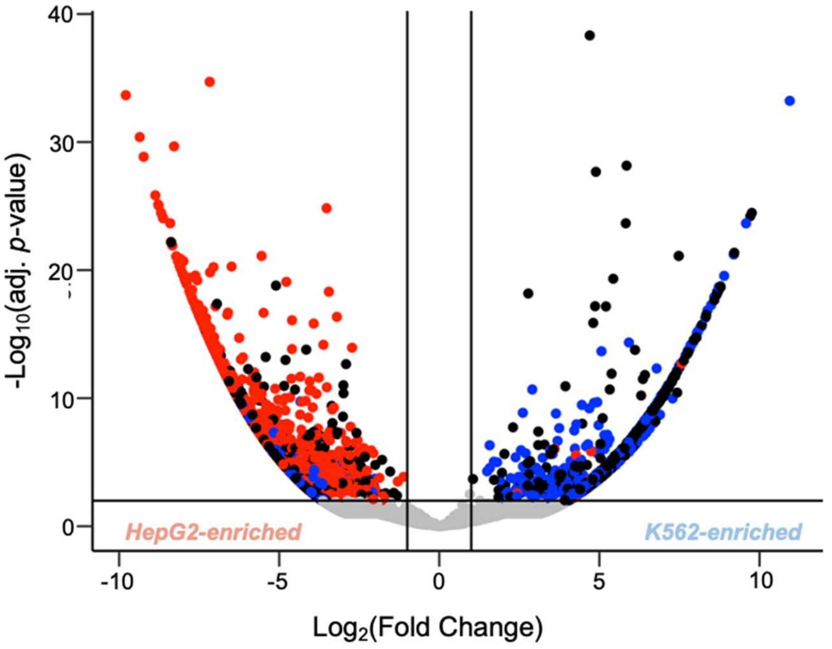

	HepG2 Loops	K562 Loops
HepG2 BG4	4378	168
K562 BG4	506	2347
Total	6598	4387

Figure S6. Cell viability assay of HepG2 cells treated with different concentrations of PDS for 24 h. The results showed that a 24-h exposure with 20 μM PDS did not confer a significant impact on the viability of HepG2 cells.

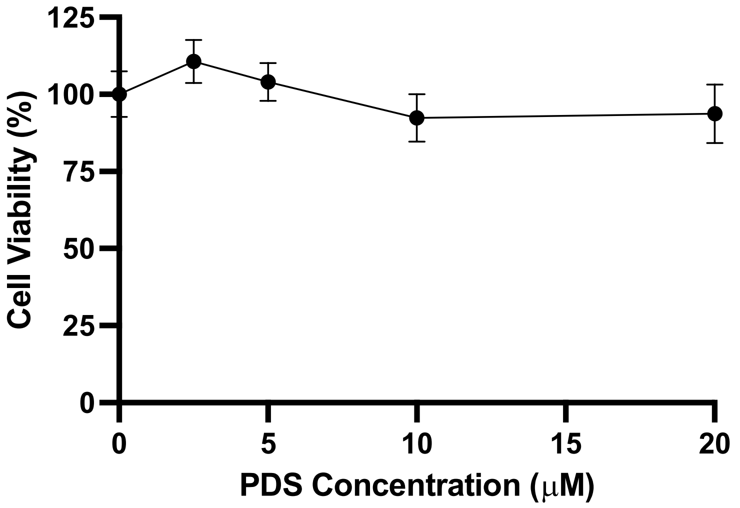

Figure S7. ChIP-qPCR experiments revealed enrichment of POLR2A at promoter and enhancer regions of *KRAS* and *MDM2* genes.

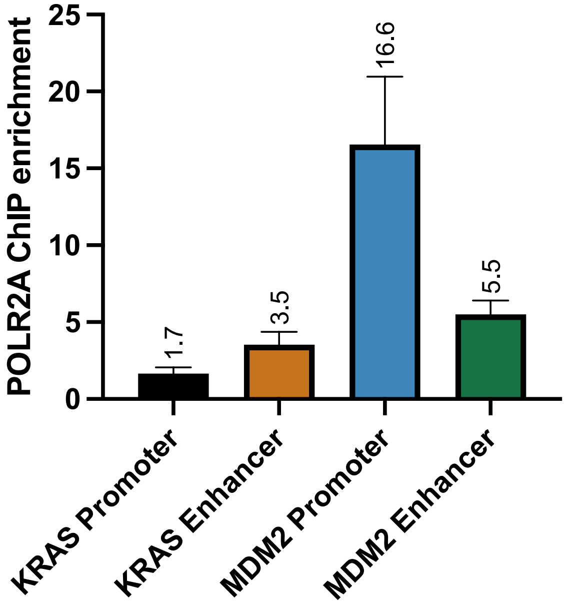

Figure S8. PDS treatment led to much more pronounced attenuation in expression of those genes with G4 structures in their promoters or with promoters being connected with remote G4 structures through RNAPII-linked DNA looping (G4) than those not associated with G4 structures (Non-G4). Two-tailed Student's *t*-test with Welch's Correction, ****, $p < 0.0001$.

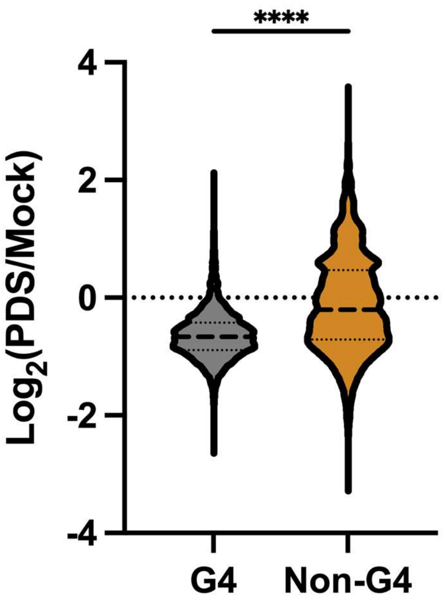

Figure S9. POLR2A ChIA-PET matrices within the regions of *AKR1C1-3* gene showing multiple long-range DNA interactions in HepG2 cells, but depleted in K562 cells, which is associated with the presence or absence of G4 structures at these loci in the two different cell lines. Panels from top to bottom: POLR2A ChIA-PET matrix in HepG2 cells, POLR2A ChIA-PET interactions in HepG2 cells, BG4 ChIP-seq in HepG2 cells, Gene annotation, BG4 ChIP-seq in K562 cells, POLR2A ChIA-PET interactions in K562 cells, POLR2A ChIA-PET matrix in K562 cells.

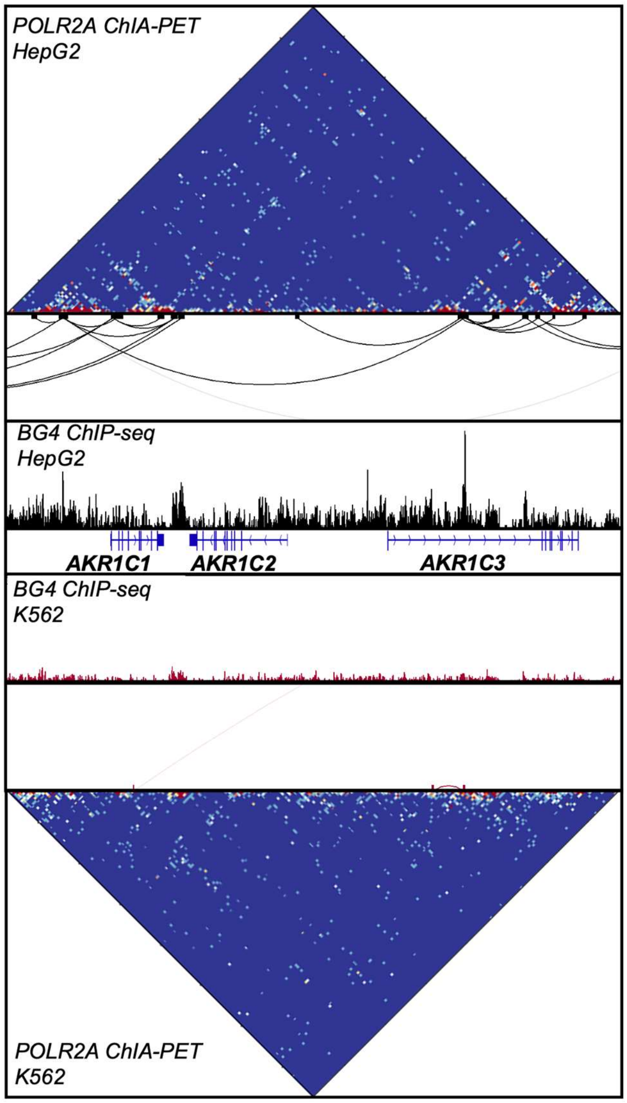

Figure S10. HiChIP interaction matrices of RNAPII in the promoter region of *AKR1C1* gene in HepG2 cells that were mock-treated (top) or treated with 20 μM PDS (bottom) for 24 h. Depicted in the middle are the RNAPII ChIP-seq tracks (middle two) revealing diminished RNAPII binding at both promoter and distal G4 site following PDS treatment.

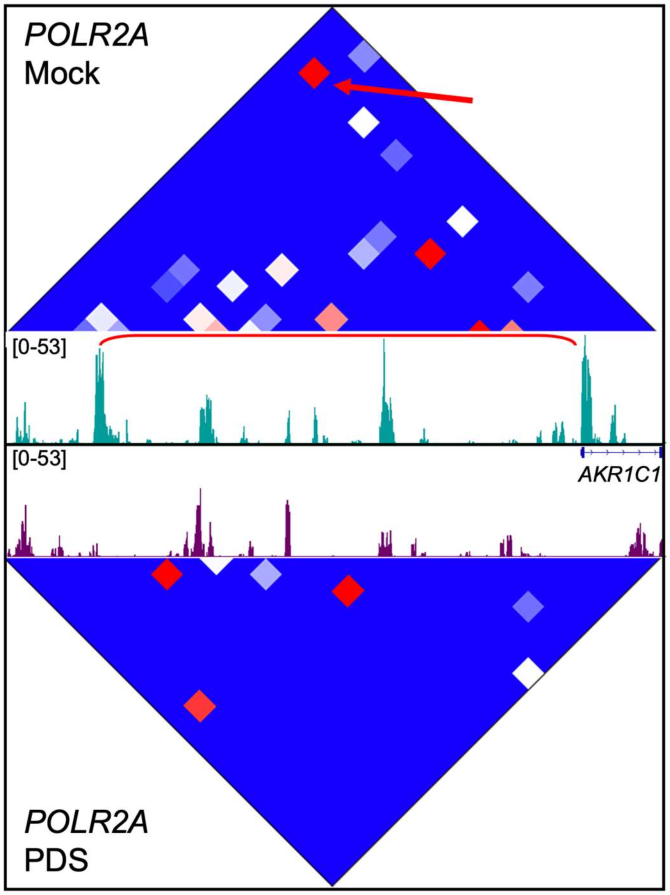

Supplement: gkad588_Supplemental_File [file gkad588_supplemental_file.pdf]
